# Clarifying differences between reviews within evidence ecosystems

**DOI:** 10.1186/s13643-019-1089-2

**Published:** 2019-07-15

**Authors:** David Gough, James Thomas, Sandy Oliver

**Affiliations:** 1 EPPI-Centre, Social Science Research Unit, Department of Social Science, University College London, London, England; 2 Africa Centre for Evidence, Faculty of the Humanities, University of Johannesburg, Johannesburg, South Africa

## Abstract

This paper builds on a 2012 paper by the same authors which argued that the types and brands of systematic review do not sufficiently differentiate between the many dimensions of different review questions and review methods (Gough et al., Syst Rev 1:28, 2012). The current paper extends this argument by considering the dynamic contexts, or ‘evidence ecosystems’, within which reviews are undertaken; the fact that these ecosystems are constantly changing; and the relevance of this broader context for understanding ‘dimensions of difference’ in the unfolding development and refinement of review methods.

The concept of an evidence ecosystem is used to consider particular issues within the three key dimensions of difference outlined in the 2012 paper of (1) review aims and approach, (2) structure and components of reviews, and (3) breadth, depth, and ‘work done’ by reviews.

## Introduction

The logic of using explicit rigorous (research) methods for assessing the research evidence in relation to a research question applies to all research questions and methods in research [1]. If reviews are ‘a review of existing research using explicit, accountable rigorous research methods’ [2] then all reviews, whether numerical or narrative, are systematic if they follow the tenets of research: of being rigorous and transparent. This applies to all levels of research whether these be primary studies, secondary analysis of primary data, reviews of research, or reviews of reviews.

In our original paper on ‘Clarifying differences between reviews designs and methods’ we argued that there is a lack of agreed terminology for different types of reviews, that the terms that did exist were not used consistently, and that there was also considerable overlap in approaches within different review types and brands [1].

We proposed that it was more fruitful to consider how reviews varied and for review authors to specify how their review is located on key attributes of aims and approaches, structure and components of reviews, and breadth, depth, and work done by reviews (See Table 1 adapted from [1]). Specifying review methods on these key attributes may lead in time to a more consistent system of terminology for systematic reviews.

**Table 1:** Key dimensions for planning reviews

**1. Review aims and approach**. (i) Approach of the review: ontological, epistemological, theoretical, and ideological assumptions of the reviewers and users of the review; (ii) review question: what the review is asking of research (and the type of information that would answer it); and (iii) aggregation and configuration: the relative use of these logics and strategies in the different review components (and the positioning of theory in the review process, the degree of homogeneity of data, and the degree of iteration of the review method). **2.Structure and components of reviews**. (iv) The systematic map and synthesis stages of a review and the potential multiple components of these stages and (v) the relation between these stages and components **3.Breadth, depth, and ‘work done’ by reviews**. (vi) Macro research strategy: the positioning of the review (and resources and the work aimed to be done) within the state of what is already known and other research planned by the review team and others and (vii) the staff, time or other resources used to achieve this	

The current paper progresses this way of thinking by situating the key review attributes within the broader contexts (ecosystems) within which reviews are produced and used. These contexts frame and influence the ways that reviews are undertaken and thus the ways that they vary. The paper first discusses the nature of these broader contexts and evidence ecosystems and why they are important for review methods. It then re-examines the three areas examined in the 2012 paper and updates them, taking account of these broader contexts.

## Evidence ecosystems: the contexts of the production and use of systematic reviews

There is a relationship between the ways in which research findings are used, how those using research engage with research, and how research is initially produced. These relationships between the use and the production of research can be seen as a system [3] or an ecosystem. A key dynamic in the ecosystem is the stance taken by users of evidence—such as those in decision-making contexts—towards the evidence that they use, evidence which is usually provided by those in academia. While traditionally, the role of an ‘evidence producer’ is seen to be quite independent of the ‘evidence user’, as users of evidence become more engaged, and invested, in the evidence that they use, so their influence on evidence production is becoming more pronounced. As the sociologist Helga Nowotny [4] asks:

Society is moving into a position where it is increasingly able to communicate its wishes, desires and fears to Science. What happens then to science as result of this reverse communication?

Signs of the demands of the changing economy of evidence use abound: from the increased focus (and demand) from policy-makers for research to have demonstrable ‘impact’, the greater interest in ‘rapid’ reviewing methodologies, the use of large-scale administrative datasets to inform decision-making, to the involvement of major IT companies in the development of new AI decision-support systems. Indeed, the traditional painstaking, lengthy, and resource-hungry approaches to systematic reviewing may appear increasingly archaic in an age where ‘evidence’ can be located at the click of a button and interventions targeted more precisely to specific recipients. In medicine, for example, researchers aim to treat patients chosen through individual genetic profiling in contrast to the aggregated population-level evidence more common in systematic reviews. In this regard, changing approaches and attitudes towards evidence synthesis can be seen as part of broader transformations in epistemologies across the academy, with ‘big data’ gaining an increasing focus and experimental research appearing more expensive, slower, and offering less precise answers in comparison. This comparison may well be unfair, as there are lively debates concerning the validity of some current applications of big data; but perceptions of ‘fairness’ are a little beside the point in the context of an ecosystem. The fact is that these new components of evidence ecosystems are being built and deployed; the question is *how* they should best be used, what role should other forms of research—including systematic reviews—play, and what methods are needed?

**Fig. 1:**
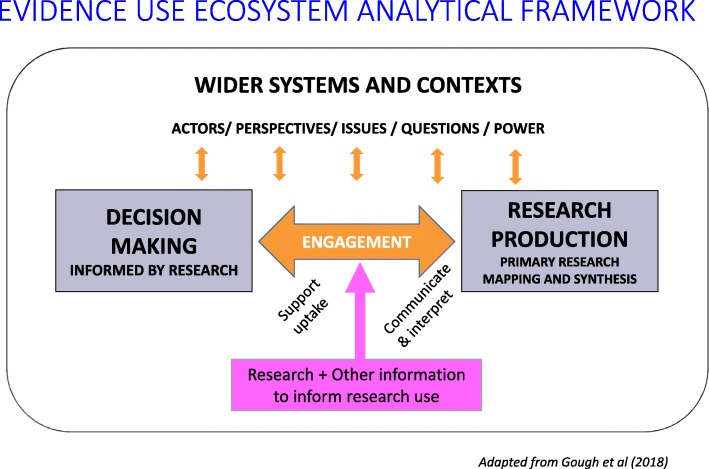
Evidence use ecosystem analytical framework [5]

The basic components of an evidence ecosystem are depicted in Fig. 1 and include the following:

- The use of research for undertaking analysis of situations and informing decisions
- Engagement of users and producers of research, providing access, and supporting interpretations of research and its uptake
- Production of both primary research and reviews of such research
- Other information used to interpret research findings
- The broader socio-political context within which the narrow evidence ecosystems of research use and research production exist

The ecosystem is more than the context within which research is produced. Ecosystems are dynamic, with different components affecting each other, either directly or indirectly, with positive and negative feedback loops. Ecosystems exist within wider interacting contexts and systems. It should also be emphasised that the producers and users of research are very varied and engage with each other in many ways. Authors of systematic reviews may, for example, be skilled methodologists and yet bring in other stakeholders such as topic specialists, likely users of a review, and broader societal perspectives to advise on the questions and methods of a review. Academics are of course themselves users of reviews of research evidence and so not all research should necessarily involve non-academic users. Evidence ecosystems exist within a broader context or broader systems within society that may influence the functioning of an evidence ecosystem in many ways. There may be structures, policies, organisations, and groups with varying perspectives (values, assumptions, and priorities) and power to actively or passively impact on an evidence ecosystem with positive or negative effects. Also, all these forces may be changing over time, and so we have the additional complexity of evidence ecosystems changing and developing within evolving wider systems.

Many authors have written about the ways that evidence systems may not function well due to a lack of coordination between different parts of the system and with the wider context within which they exist. As well as all of the political factors determining how people act in relation to research and evidence [6], there are many other factors that affect the efficient functioning of an evidence ecosystem:

- *Functional engagement*. Potential users of research not being aware of research or not having the capacity, motivation, or opportunity to make use of research in their work [7] and researchers lacking the structural mechanisms, skills, or motivation to engage potential review users when shaping review questions [8, 9]
- *Alignment of perspectives*. The research available being driven by academic interests (a ‘push’ production model) and not being relevant in the topic or focus for potential users of research (with a ‘pull’ demand and problem-solving model) [10]
- *Consideration of the full evidence base*. Individual studies being used to inform decision-making that may not be representative of the broader evidence base [2]
- *Quality and relevance standards*. The research not being of sufficient quality or relevance [11]
- *Coherence of policies, structures, and processes***.** Across the different components of the evidence ecosystem [5]
- *Balance of resources***.** Available to different parts of the evidence ecosystem [5]
- *Power and/or coordination***.** With the other organisations and systems within the particular area of public policy [6]

A common example of imbalance in the investment of resources is the relatively small amount of funds spent on research on public services compared to the provision of those services. Probably only a fraction of policy and practice decisions are informed by reliable, up to date research evidence and only a fraction of relevant evidence is implemented in changes to policy and practice. This reflects insufficient exploitation of research knowledge and produces sub-optimal decision outcomes that undermine the welfare of citizens; it is therefore wasteful of both investments in research and in service resources. There is also waste from research being poorly undertaken, poorly reported or not necessary as it has not taken into account (from systematic reviews) what has already been studied [12]. Another example is the high proportion of resources spent on primary research compared to the review and synthesis of that research, let alone enabling the use of such evidence bases in policy and practice decision-making. A further example is the inefficiency in the curation of research knowledge described later in this paper.

Growing awareness of research use and research production (as related parts of evidence ecosystems) has led to attempts to study and intervene in these systems. This has included financial drivers to encourage research use, as in rewards for research impact on UK universities, or career incentives where use of research is recognised in reviews of performance. The development of knowledge-brokering individuals and organisations facilitates two-way knowledge exchange.

Indeed, ‘research on research use’ is developing as a field of study that addresses the role of evidence in policy, practice, and individual decision-making. This field spans efforts to increase user participation in research [4] (see later in the paper), enabling research evidence to be considered in decision-making, and understanding and enabling the mechanisms and behaviours by which this can happen. One very particular subset of this work is implementation science which is the study of methods to promote the systematic uptake of research findings and so tends to involve more of a one-way ‘push’ model of research evidence determining decision-making.

Taking a systems view of research use and research production also has implications for understanding the role of systematic reviews. In particular, the following:

1. Clarity and consensus about definitions, aims, and social values
2. Relevant questions and relevant evidence for decision-making (including academic decision-making about future research)
3. Data and research infrastructures
4. A dynamic temporal approach to reviews

These four issues are discussed in the next section in terms of the aims and approaches of reviews. This is then followed by comments about the implications of an evidence ecosystem approach for the other two main attributes of reviews of structure and components of reviews, and breadth and depth of reviews.

## Review aims and approaches

### Clarity and consensus about definitions, aims, and social values

At the core of the evidence ecosystem illustrated in Fig. 1 is a mutual exchange of ideas between people making policy, practice, research, or personal decisions and researchers producing relevant evidence. Where this exchange starts—with decision-makers seeking evidence, researchers offering evidence, or a mix of the two—varies. The purpose of the exchange is to achieve clarity and consensus about what the research needs to address and what the findings mean, that is, clarity and consensus over the concepts at the core of the work—what they are, how they are defined, and how important they are (in other words, the social values influencing the work—though there are of course a range of other motives also at play such as tactical and political use of research and academics’ wish to promote their work) [6, 10].

We have previously studied this engagement for the production of policy-relevant reviews [8, 9] and draw out the implications here. Where the research literature offers both clarity and consensus about the definitions and importance of key concepts, it provides a sound starting point for supply-driven reviews. Where such clarity or consensus is lacking or is contested by stakeholders beyond the research community, engaging stakeholders early in the review process is crucial.

This analysis expands the review methods from a technical analysis of the literature alone, to include methods for how researchers and research users engage with each other. It has major implications for the supply and demand of evidence. Weiss [10] characterised the supply of evidence as the knowledge-driven model and the demand for evidence as the problem-solving model. In supply-driven reviews, researchers largely justify their focus by drawing on the existing literature and then engage with potential research users through intermediaries who coordinate the peer review process; sometimes, they also engage with stakeholders using a steering group to set the focus. In contrast, in demand-driven reviews, the researchers invest more resource early on in understanding the problem being faced by specific research users and how it is understood, not primarily by the existing literature but initially at least by specific decision-makers; they do this by engaging directly with specific research users, sometimes supported by knowledge brokers or by adopting knowledge-brokering practices.

The perspectives and social values to shape supply knowledge-driven reviews come largely from researchers and the existing research literature. For demand-driven, problem-solving reviews, they come largely from the potential users of the reviews. An example of the latter comes from NICE, who undertake reviews on cost-effectiveness of services in order to make utilitarian value decisions about resource allocation of different services to different patient and societal groups. The social values that shape these reviews come from NICE’s social values policy and stakeholder groups convened to develop guidance for practitioners and from public consultations with stakeholders [13].

Research also has implicit social values of the importance of rigour and transparency in research. There may then be further values that research should attend to, such as the relevance of research to sub-groups in society and the impact of our understanding and response to societal inequalities (as in, for example, Cochrane’s approach to inequality and research) [14]. A review of social values in the development of health and care guidance health services identified very many social values referred to in the literature (Table 2) and there may be many further social values in other areas of social policy.

**Table 2:** Social values related to the development of health and care guidance [13]

1. Utility and Efficiency 2. Justice and Equity 3. Autonomy 4. Solidarity 5. Participation 6. Sustainability 7. Transparency and Accountability 8. Appropriate Methods of Guidance Development	

This portrayal of the production and use of systematic reviews as a social enterprise, not only as a technical enterprise, is underdeveloped. Details of interpersonal communication between policy-makers and researchers in shaping systematic review questions and conceptual frameworks [9] are given little attention in methodological research and guidance, other than noting the value of engaging stakeholders [8]. Interpersonal communication in guideline panels is similarly given little attention other than panel membership, despite the wider literature on collective decisions about technical issues revealing the influence of discussion time and skilled facilitation on decisions [15]. Yet analysing existing methods for engaging stakeholders reveals a clear rationale for choosing between these methods [16]. When the meaning of key concepts is clear and agreed in advance, as with supply-driven reviews grounded in academic literature, small numbers of stakeholders are sufficient if drawn from key organisations and engaged through membership of an advisory group or formal consultation. In contrast, when the prior meaning of key concepts is unclear or contested, as with demand-driven problem-solving reviews, larger numbers of diverse stakeholders are required to be engaged in constructive discussion to create meaningful questions and conceptual frameworks and to draw out the implications from the emerging findings.

### Relevant questions and relevant evidence for decision-making

#### Theory, complexity, aggregation, and configuration

**Fig. 2:**
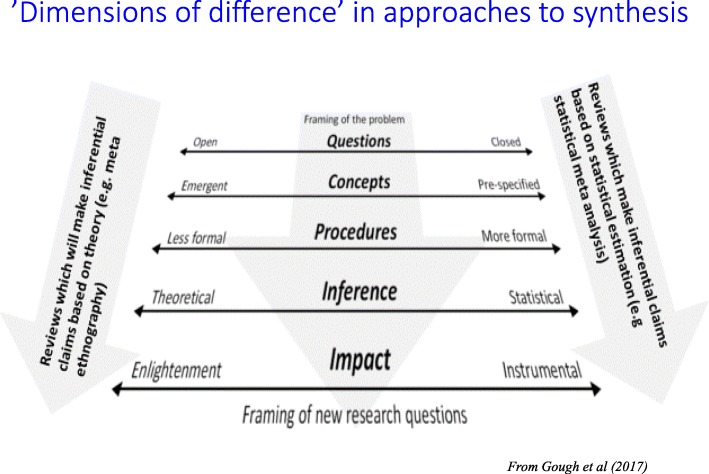
‘Dimensions of difference’ in approaches to synthesis [2]

A key tension in science for centuries has been the extent to which knowledge can be based on observation of the world alone, and how much depends upon people’s ability to theorise about these observations [17]. This balance between observation and theory (or empiricism and rationalism) underpins the differences we see between different approaches to reviewing. Running alongside an emphasis on observation tends to be the importance of replicable scientific methods, the pre-specification of concepts, and statistical inference (Fig. 2). Reviewers—and hence, review methods—who emphasise the importance of theory, and the development of knowledge through the organisation of concepts and ideas, tend to favour more exploratory and emergent methodological approaches, where the purpose of the review is to develop new concepts and theories and where the basis of its inference is theoretical, not based on statistical prediction.

Cutting across the above concerns is the degree to which a review aims to understand and explain differences between study findings, and the degree to which it aims to gain more precise estimates of, e.g. the effect of an intervention. Statistical meta-analysis is a good example of the latter approach, where the findings from multiple studies are *aggregated*, and a combined measure of effect is obtained. This contrasts with review methods which aim to understand, e.g. what is causing interventions to have different effects in different settings, or to explain why different people have quite different understandings of a given phenomenon. Such reviews *configure* the findings from the studies they contain, comparing and contrasting them with one another and analysing patterns in the data.

Reviews which aggregate or configure their findings cannot be aligned neatly according to whether they emphasise observation or theory/empiricism or rationalism. While a statistical meta-analysis which compares, for example, the relative effectiveness of two drugs may well contain relatively little configuration and place relatively little importance on the theory of *how* the treatments work or whether they work better for some groups than others, most other types of review tend to have elements of both configuration and aggregation. For example, another statistical method that can place great emphasis on observation, and relatively little on theory, is a network meta-analysis (NMA—see below), which compares a range of treatment options for a given condition with one another. An NMA places high importance on analytical methods for *configuring* study findings.

Early development of review methods in the 1990s focused on the meta-analysis of randomised trials, as exemplified by the Cochrane Collaboration. While this approach has proved highly relevant for answering some questions, there has been a gradual increase in interest in reviews which do more than estimate the effect of a single intervention and that are more aligned with other needs of their users. The need of review users to understand what causes differences in intervention effects has driven increased interest in review methods that utilise theory to enable them to explain the causal processes by which an intervention has its effects. The theories developed about causal processes can assist with both improving the efficacy and the applicability of policies and services. Some of these methods operate through extensions to statistical meta-analysis using a priori techniques such as the use of logic models to analyse data to undertake finer-grained tests of theory [18–20]. Some of these methods, such as qualitative comparative analysis (QCA), have been more concerned with developing theory and examining patterns of factors associated with positive and negative outcomes [21]. In addition, there has been greater use of multicomponent mixed methods reviews to combine the results of studies that both test and generate theory, and interest in complexity science and systems theory to better understand the interactions between an intervention and the context within which it is introduced [22, 23].

While there are many more developments to review methodology than have been mentioned here, the key point to observe is the way that these new methods are *emergent* properties of the evidence ecosystem. The dynamic interchange of ideas between users and reviewers has led directly to a quite substantial shift in emphasis and methodological practice in a relatively short period of time.

#### Broadening the types of evidence: administrative, cohort, and individual participant data

While most systematic reviews bring together the results of published reports of studies, some reviewers seek out the raw results data from trials and meta-analyse these instead in ‘individual participant data’ (IPD) meta-analyses. Such an approach has many advantages, including data quality (such as dealing with missing data), more precisely matching data across studies (such specific time-points for data collection), the investigation of sub-groups and addressing bias [24]. Collating and analysing these larger datasets takes longer and demands more specific expertise, than using the summary findings of studies reported in publications.

Different primary research and research synthesis questions require different methods, data, and forms of analysis. The 2012 paper [1] discussed this variation and in particular the balance between aggregative and configurative logics of synthesis. A particular development in the last few years has been the awareness of the value to decision-makers of administrative data in association with experimental and process data. Administrative and cohort data can do the following:

- Provide information on prevalence
- Identify correlations that can inform theory development and testing
- Monitor performance over time including providing outcome data for experimental trials

Such data may be used by policy and practice instead of synthesis of the findings of experimental studies. They can also be used in combination, where synthesis of experimental data can inform decisions, the outcome of which can be monitored by administrative data. In these situations, the availability and use of IPD can greatly enhance such analyses, as more fine-grained information about participants can be utilised. Also, local data can be calibrated against other areas or national data to assess local services (informed by national or locally focused synthesis). The use of these new datasets is not without its challenges, however, and the current heightened interest in the use of big data for decision-making may carry with it the risk that important principles of research are forgotten [25].

The mixing of administrative and experimental data is a good example of how user-driven research can lead to a broader view of relevant research, though more methodological development is needed here [26]. Synthesis is not simply the bringing together of research defined by research method. Synthesis is the bringing together of the relevant research that helps to address the question being asked.

#### Interpreting research to inform decision-making

Research findings have little meaning on their own; they are interpreted first by researchers reporting their work, then by readers (whether researchers or other users) who bring their own perspectives and understanding of the context to which the interpreted knowledge is to be applied.

Examples of such other knowledge could include the following:

- Prevalence data (how frequent an issue is it?)
- Informal (non-codified) knowledge
- The perspectives of other important actors (what do others involved think?)
- Resources (available for any change)
- Other contextual information (that may affect decision-making)

Some of this other information may itself be the product of research, such as prevalence data or surveys of user views about the acceptability of different courses of action or the effects of certain situations. Figure 1 shows that research can provide findings relevant to decision-making, but then further forms of research can provide extra evidence to inform how research is interpreted. For all of these different forms of research, there is the issue of evidence standards—what are the requirements of quality and relevance for research evidence to inform decision-making?

#### Evidence standards

If research evidence is to be relied upon to inform decision-making then there needs to be clarity about the standard of evidence that is being applied.

For synthesis, the evidence standards are the quality and relevance of [11] the following:

1. The method of undertaking the review (suitability of method + standards achieved + relevance to the review’s focus)
2. The studies included in the review (suitability of method + standards achieved + relevance to the review’s focus)
3. The totality of evidence produced (nature of the totality of evidence + extent of that evidence)

Different research questions, different research methods, and different parts of the evidence ecosystem involve different evidence standards to justify these claims. Some formalised evidence standards methods and criteria have been developed for some of these issues. There are reporting standards that specify the main methods components that should be reported for some types of review [27, 28]. There are also systems for the critical appraisal of either the methods of review [29, 30] or the studies included and the totality of evidence in the reviews [31, 32]. The complexity of clarifying and being transparent about evidence standards is becoming even more challenging with more complex research questions and methods and with multicomponent reviews that address a number of different sub-questions. However, even for straightforward ‘what works’ questions, there are many aspects of the evidence ecosystem where evidence standards are not clear or coherently or consistently applied [5, 33]. This need for coherence, consistency, and complexity of evidence standards applies to both the main research being used to inform decision and to the extra evidence being used to inform interpretation of that research (as in Fig. 1).

Although a minimum level of quality and relevance is required to make any defensible evidence claim, the extent of quality and relevance required will depend upon the nature of the decision being made. The evidence needs for a decision about how to make a very immediate pressing decision where there may be a choice between using limited evidence or not using research evidence at all -  can be different to a more measured less time-pressured decision-making process. For all of the many types of review question and types of systematic review, there can be many levels of evidence standards for making different evidence claims based on those reviews.

### Data and research infrastructures

#### Current knowledge curation systems are insufficient

Reviewers spend significant amounts of time undertaking tasks which essentially involve making up for failures in current global systems of research knowledge curation. First, the early phases of a systematic review are dominated by the work involved in locating relevant studies. The reasons that such extensive searching—and subsequent sifting—is needed are because systems for indexing research are not fit for purpose, i.e. they fail to ensure that research can be located reliably and efficiently. Research is often published in journals—many of which are not indexed systematically, and where they are indexed, the indexes are distributed across multiple databases, some of which lie behind subscription-only paywalls. Most research is not catalogued using widely used controlled vocabularies, such as Medical Subject Headings (MeSH), but even if it were, the controlled terms used do not enable research to be located accurately, and sensitive free-text searches are normally necessary (in addition to searches that use MeSH).

Second, if on average, approximately 3,000 studies are screened for inclusion for each systematic review (including updates of reviews), and in health care alone over 25,000 reviews are published each year, then more than 75 million citations were screened for inclusion in systematic reviews published in 2017 alone (and, as many will have double-screened citations, the number will be far more than this). This is equivalent to more than three times the entire contents of PubMed being manually examined by systematic reviewers every year: a colossal investment of resource and, bearing in mind the fact that the same studies will be examined multiple times, a massive duplication of effort.

Third, the later stages of a systematic review involve the laborious ‘extraction’ of data from PDF documents into structured templates, often requiring the manual transcription of statistical data. Such processes are time-consuming and error-prone, resulting in the need for independent extraction of data by two or more researchers to minimise errors.

The inefficiencies identified above stem from the fact that research publication has traditionally been disconnected from the subsequent utilisation of the published research. Journals were essentially a means for academics in the same community to communicate with one another—rather than the means through which research knowledge moved from academia into being used to inform policy and practice. As demand for research utilisation has increased, so the deficiencies in the current research-publication ecosystem have become exposed. This includes, for example, the increased interest in open science to enable free access to publicly funded research rather than the work being hidden behind a paywall of commercial publishers. A more functional ecosystem would ensure that research was properly curated, in ways which made it easy to find, access, and re-use. As in other areas, while these issues are felt acutely in systematic reviews, they are experienced elsewhere too, and widespread dissatisfaction with current practices is beginning to result in systemic change.

#### Data integration and sharing

An ecosystem that supported more efficient systematic review workflows would ensure that duplication of effort was minimised, rather than the standard. In order to achieve this, review data need to be shared so that the decisions made about a given study in one review are available to other reviewers. This is relatively easy to accomplish when the users are part of the same organisation, though it does require consistency in the use of tools and classification schema. Cochrane and the National Institute for Health and Care Excellence (NICE) are two organisations which are moving to systems which facilitate the (internal) re-use of review data. The Systematic Review Data Repository (SRDR) is another initiative that aims  to facilitate the re-use of review data. However, while these steps may contribute significantly to individual organisational efficiency and some limited data re-use, they do little to change global practice; for better efficiency at an ecosystem level, we require systems—and most importantly, consistent classification schema—to be deployed and used across organisations.

Cochrane has developed, and is advancing, an ‘ontology’ for Population, Intervention, Comparator, Outcome (PICO) codings to be used as just such an ecosystem-level schema. The PICO schema models the key characteristics of a controlled trial, accounting for relationships between concepts, and it contains thousands of terms from established controlled vocabularies, such as Snomed CT, MeDRA, and MeSH. The fact that these terms are embedded within the PICO model and selected specifically to describe the studies included in systematic reviews, makes them far more powerful as a knowledge curation device, than for example, standard MeSH headings. Systematic reviews of effectiveness can be specified precisely using their PICO classifications, making it possible to predict which trials should be included using the same classification schema (assuming the studies for inclusion have also been so classified). Organisations which use this schema to describe their included trials will be able to share their data with one another, benefitting from the classification activities undertaken by other organisations and contributing the classifications which they have assigned for others to use.

#### Moving data curation activities ‘upstream’

Most systematic reviews (and downstream users of review findings) are concerned with a subset of papers published and indexed in databases. For example, while journals publish many editorials, book reviews, commentaries, and case reports, such papers are of limited interest to systematic reviewers; they are often retrieved in searches and need to be manually sifted out of the pool of potentially eligible studies. Unfortunately, due to the lack of precision with which studies are indexed, there is little that can be done about this within the confines of individual reviews. At an ecosystem level though, it is possible to conceive of systems which account for some of this deficit and prospectively identify the types of research relevant for use in systematic reviews. For example, Cochrane has been identifying reports of randomised controlled trials (RCTs) for more than two decades. It has assembled a large database of such trials and, in some areas, can reasonably claim to have all the trials ever published. When such comprehensive datasets exist, the task of study identification for inclusion in reviews is greatly simplified compared with standard practice: instead of undertaking extensive, sensitive searches of multiple databases, authors need only to search a single source, confident not only that they need search nowhere else, but also that a relatively few papers will be retrieved for checking, because only RCTs will be returned.

Recognising the huge efficiencies that a comprehensive database of RCTs can bring, Cochrane has now developed infrastructure and processes to identify RCTs as soon as they are published (either as reports in journals or as records in registries of clinical trials). This work involves the regular searching of multiple databases, and the systematic identification of all RCTs retrieved. While not yet adopted as global practice across the systematic review world, these activities point the way towards a radically different systematic review process, where some labour is shifted outside the context of specific reviews and invested instead, in upstream knowledge curation activities [34]. All of this of course depends on full reporting of trials so that they can be included in these systems [35].

#### Information technologies, automation, and artificial intelligence

The final component to consider in this section on the changing research infrastructure environment is the explosion in the use of information technologies to streamline review processes. Systematic reviews may now include a combination of human *and* machine reviewers, with machine learning technologies assisting in activities such as citation screening, risk of bias assessment, and even synthesis. Indeed, the ambitious Human Behaviour-Change Project (HBCP) aims to locate vast quantities of relevant research in order to test the ability of machine learning and reasoning algorithms to predict effect sizes for particular combinations of behaviours, interventions, populations, and settings automatically [36]. Full automation of the entire review process would probably be a step too far for most systematic reviewers, but there is now a widespread recognition of both the need to automate some aspects of the systematic review process and of the fact that it is possible to do this in methodologically sound ways.

The use of automation in systematic reviews is in its infancy, and tools and methods are under constant development and evaluation. It is clear, however, that effective machine learning often relies upon high-quality data from which to ‘learn’, and that systematic reviewers will need to be involved in the development and validation of such ‘training’ data sets. A good example of this practice is the Cochrane Crowd citizen science platform, where a community of volunteers undertakes knowledge curation tasks that contribute to the development of high-quality datasets. As well as being invaluable for systematic reviews, these datasets are also ideal for machine learning, and a high-performing machine learning classifier is now available, able to distinguish between reports of RCTs and non-RCTs. Tool development is also underway that can assign PICO classifications automatically.

When machine learning/AI technologies are put together with the existence of shared classification schema (such as the ‘PICO’ model above) and the recognition that systematic review effort can effectively be deployed in ‘upstream’ knowledge curation work, we can begin to envisage a radically different systematic review workflow. Instead of undertaking extensive, sensitive searches, an author of an impact review can simply specify their PICO question in a repository of all relevant trials which have been systematically classified according to the PICO schema [36]. They may also find that another reviewer (whether human or machine) has already undertaken the necessary data extraction and risk of bias assessment of their included studies and their review work involves the selection of outcomes and interpretation of findings. As well as supporting the creation of new reviews, such data structures and workflows lend themselves ideally to the ‘surveillance’ of research and thus to the early identification of new studies which might be included in existing ‘living’ systematic reviews and guidelines [37, 38].

While this appears to be a radical departure from current practice and certainly requires the development of new tools, practices, and reporting standards, the nature of the final synthesis product may not be all that different to what is currently known. The real departure may be in the domains and the types of questions and studies which are able to avail themselves of these efficiencies. It is notable, from the above examples, that these moves are being driven in the health sector and are focused on RCTs. This may privilege particular questions and epistemologies over others and, while the danger of this is not a good reason to abandon the above efforts, members of the ecosystem with the ability to determine resource allocation may want to address this epistemic inequality.

#### Evidence-informed recommendations and guidance

There has been considerable investment of effort in the processes for systematically reviewing research literature. There is also the development of systems and infrastructures for research evidence to support decision-making. Some of this is simply making research evidence available in web portals and toolkits. Another is formal processes for interpreting research evidence in order to make recommendations or guidance for policy, practice, or individual decision-making. One example is structured processes for considering various forms of research evidence to making recommendations on health interventions as with GRADE and Evidence to Decision frameworks [39]. The aforementioned move towards asking broader questions and engaging with complexity has led to initiatives that seek to integrate research evidence, complexity perspectives, and the norms and values of decision-makers [40]. This can include deliberative processes such as in the development of evidence-informed practice guidance by the English National Institute for Health and Care Guidance. NICE guidance committees consist of a range of relevant stakeholders that commission systematic reviews of the relevant research and then interpret this evidence in terms of their perspectives (values, assumptions, and priorities) and their knowledge of the context within which the decision is to be made. Such work highlights the increasing strength of connections in the evidence ecosystem between research production and use.

## Structure and components of reviews

Methods of systematic review have become increasingly complex. This may be partly because of technical developments in review methodology, but as mentioned above, it is also driven by increasing awareness of how reviews can be used. In other words, the changes in review practice that we see—towards broader reviews and in the use of theory and complex questions—can be understood as the outcome of feedback loops in a more connected evidence ecosystem.

### Multicomponent reviews

One way in which reviews can be more relevant to use is by the reviews asking broad questions. This can be achieved by the review asking a number of sub-questions that can be addressed sequentially or in parallel. These multicomponent reviews are sometimes called mixed methods reviews though the mix of methods can arise in at least four ways:

- *Mixing of components in the overall methods of review*. These are the differences between components of the review and how these are mixed together sequentially or in parallel. A realist synthesis, for example, may have a first component configuring a mid-level theory followed by a second stage where multiple aspects of the theory are empirically tested in a number of parallel sub-components [41].
- *The mixing of review methods within individual components of the review*. So, for example, the methods by which a mid-level theory is empirically tested in different stages of a realist review.
- *Mixing of methods of primary research across the different review components*. As the review components are addressing different sub-questions with different review methods, then they may also be considering different types of primary studies. In statistical meta-analysis, however, the a priori aggregative testing of a hypothesis may be followed by a post hoc configuring of data from the same studies.
- *Mixing of methods of primary research included within individual review components*. This may be less likely to occur as one of the reasons for splitting a review into different components is to avoid considering very different types of primary studies. In realist reviews, however, the empirical testing of a mid-level theory may involve all sorts of research (though these could be described as further sub-components of the review).

Such use of broad-based reviews with multiple components is also relevant to the issue of the ambition and work done by a review. A multicomponent review can be a little like undertaking several reviews together (see the “Breadth, depth, and ‘work done’ by the reviews” section at the end of this paper).

### Living reviews

Mention has already been made of living reviews that provide a continually updating resource within an evidence ecosystem. While these may not necessarily change the procedures by which reviews are undertaken (apart from the frequency of update), they do change the way that reviews are thought about—and understood as ‘living’ reviews [42]. Firstly, they move the focus of research knowledge to the review rather than on primary research. Users are likely to want to know what the evidence base as a whole says and do not always need access to primary research (though this of course should be available). When new primary research is reported in, for example, specialist or general media, the main issue should be what the new research contributes in the context of what is already known. Commonly, however, research is reported as individual primary studies existing in isolation. Similarly, for researchers undertaking primary research, the starting point for considering what research is required is a knowledge of what the current evidence base is and how new research might change that evidence base. An example is a power calculation to determine the necessary sample size to test for evidence of the effect of an intervention on certain outcomes in a randomised controlled trial. A review level perspective suggests that the power calculation should be based on the sample size necessary for the outcome to change the conclusions of the existing systematic review findings (often by providing a more precise estimate of effect and reducing uncertainty) [37, 38]. Secondly, as discussed above, seeing a review as a continually updated product has implications for the automation of methods to identify and include the findings of new primary studies [38].

### Mixed treatment comparisons/‘network meta-analysis’

One area of ecosystem evolution in the past few years that has had a marked effect on the structure of systematic reviews has been an increased focus on comparing the relative effectiveness of different interventions with one another in the same analysis. Ironically, the type of systematic review traditionally known as a ‘what works?’ review rarely answered this question; it focused more on ‘does it work?’—i.e. the effectiveness of a specific intervention. This was often a poor fit with decision-making contexts in which users of research were genuinely interested in the comparative issues that answering a ‘what works?’ question implies (e.g. in determining the most appropriate treatment from a range of options). In response to this ecosystem dynamic, the methods for network meta-analysis have evolved and are now considered a ‘core’ part of Cochrane methods (the new Version 6 of the Cochrane handbook now has a major new chapter on network meta-analysis, placed in the ‘core methods’ section of the book) [43]. While requiring a little more specialised expertise (and time) to conduct than systematic reviews answering narrower questions, the method of network meta-analysis can be considered an extension of pairwise meta-analysis and one which should be considered when the question requires the formal comparison of multiple interventions with one another [44].

### Maps, maps of maps, reviews of reviews, and scoping reviews

Investigating existing studies is important not only for synthesising their findings, but also for navigating bodies of literature. Describing studies without synthesising their findings (to create maps) helps identify bodies of literature where synthesis would add value and gaps in the literature where primary research would add value. Describing systematic maps (to create maps of maps) identifies bodies of literature that have and have not been ‘mapped’ and so provide a navigation tool. It is important to note that some people sometimes refer to various forms of systematic (and non-systematic) maps as ‘scoping reviews’. The current authors use the term scoping in a different way to refer to an overtly non-systematic snapshot view of the nature of an evidence base (what studies have been undertaken and what they conclude). These can be useful in planning a systematic review. This principle of pulling together broad literature is sometimes applied by systematically reviewing systematic reviews and thus creating reviews of reviews. Here, we consider some of the different approaches to mapping.

Systematic reviews commonly have stages of review question, conceptual framework, inclusion criteria, search strategy, screening, coding of information from each study, quality and relevance appraisal. and synthesis of study findings to answer the review question. Systematic reviews usually have an implicit or explicit mapping stage where the coding of information from each study is used to describe the research field (as defined by the review question). In many cases, a map is important to inform the synthesis as in, for example, having a broad mapping question and then the inclusion criteria are tightened in order to undertake a narrow synthesis on a subset of the map [45]. But maps can also be important products in their own right. They can indicate what research has been undertaken—and how—and can help indicate what further research might be useful (sometimes referred to as ‘gap maps’). The methods for undertaking a map are exactly the same as for other forms of systematic review, but the process stops with the description of included studies. The difference may then be in why and how the studies are described.

**Fig. 3:**
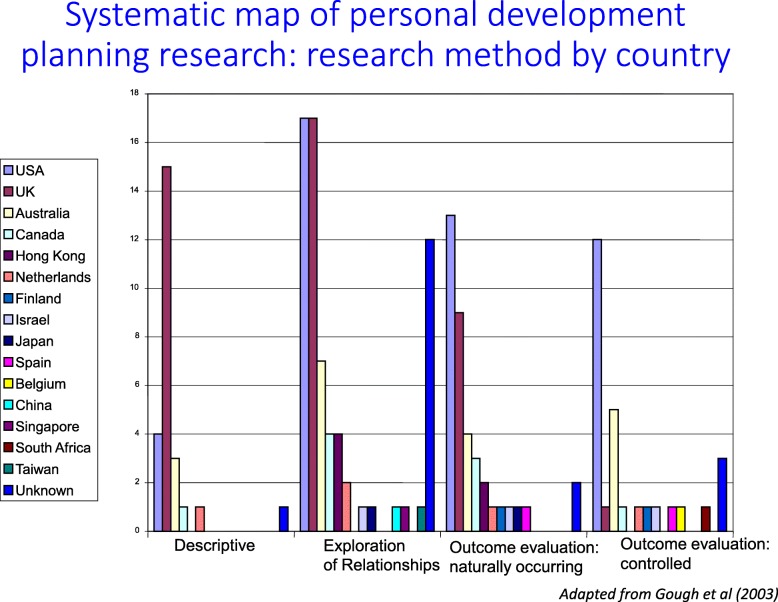
Map of personal development planning research: research method by country [47]

The EPPI-Centre has been undertaking maps as separate products since 1996 [46]. What aspects of studies are described in such maps depends on what is of interest to the authors of the map. The variables coded about each study may include such things as the aims, methods, concepts, and measures used in the studies. Maps can be on any research question with any inclusion criteria, but if they happen to concern impact questions, then the mapped variables often include PICO classifications as well as the theory of change and process measures. Figure 3 shows an example of one part of a map on personal development planning (PDP) from 2003 showing how the research methods of included studies varied between the UK and USA [47]. Visual presentations are very useful for communicating map results, but this is only an issue of presentation and does not define what maps are: the systematic identification and description of pre-existing research. Databases can also be a useful way of presenting maps as a product. A follow up of the EPPI-Centre PDP map coded included studies on 37 variables that was made publically available for those wanting to know what studies had been undertaken and how.

Maps can contextualise research projects within policy and practice issues and inform priority setting for future research [1, 2, 45]. Taking an evidence ecosystem approach focuses attention on what users of research would want to know from maps and how this feeds into map production and then to future primary research. As maps have become more popular as products in their own right, the issue arises of mapping maps to clarify what maps are available and so create maps of maps.

As systematic synthesis reviews become more common, so do maps of such reviews. Syntheses of previous reviews (reviews of reviews) are also of growing interest. They create challenges from the  existing reviews having different inclusion criteria and methods. Overlaps in criteria can also mean partial duplication of studies and thus partial duplication of findings into the review of reviews. There may also be gaps in coverage from existing reviews that can be filled  by new reviews. Such mixes of overlapping reviews with different inclusion criteria may make synthesis challenging. However, they can still provide a holistic overview of research activity and findings and how these vary with context [48].

Despite this expansion in review types, much of this work is still driven by a relatively narrow view of research questions and methods. Maps and reviews of reviews tend to be focused on narrow impact questions and structured around four core elements of PICO. There is considerable opportunity for a more varied landscape of maps and reviews of reviews asking questions about generating and exploring, rather than only testing, ‘black box’ hypotheses.

## Breadth, depth, and ‘work done’ by reviews

There is a tendency to see systematic reviews as being fairly homogeneous in terms of being of a particular breadth, depth, time taken, and the resources they use. This is clearly not the case. In addition to the range of different types of review questions that reviews can address, reviews also vary in the following:

- The extent that the review question is broad or narrow
- The complexity of the question and of the method used to address it (including multicomponent reviews)
- The degree of detail in which the questions are addressed
- The degree of rigour of the review methods

Reviews therefore differ in their ambition, their time and cost, and the resultant amounts of ‘work done’ [45]. This is not controversial in primary research where studies vary considerably in size and ambition. In terms of systematic reviews though, the issue of ‘work done’ is clearly shown by the large scale of some multicomponent reviews which ask broad questions with sub-questions addressed by different review components. Rapid reviews tend to be at the other end of the continuum are rapid reviews with fewer resources and with less ‘work done’

### Rapid reviews

An area of high interest and tension in the evidence ecosystem concerns ‘rapid’ reviews. Decision-makers are naturally attracted to an approach that promises to deliver the evidence more rapidly, but this is not without its challenges. The ability of reviewers to deliver their syntheses more quickly often requires some trade-offs. For example, increased rapidity may be achieved in various ways such as the following:

- Narrower review questions
- Limited depth of examination of the review question
- Reducing the extensiveness and rigour of the review methods
- Additional resources of staffing or automation
- Easier review questions such as those with pre-agreed concepts
- Umbrella reviews that use already existing reviews as their main source of data

Reducing the breadth of the review question and framing the review question narrowly may provide a quick and useful product, but there may be dangers of the review findings being too narrowly proscribed to inform decision-making. Similarly, a rapid review can save time by being limited in depth by, for example, only collecting small amounts of data on each included study. The review question may seem the same, but the data and the resultant analysis may be less detailed. The review can also save time by reducing the rigour of the method. One example of this is a more limited search strategy which increases the risk of relevant primary studies being overlooked. Other examples include having fewer stakeholders to engage and comment on the review, fewer discussions and less learning and iteration as the review progresses, and reducing the amount of internal quality controls such as checking the coding and screening of studies.

There are also other ways in which rapidity can be achieved without necessarily reducing the breadth, depth, or rigour of a review, and there are other cases where a review may be rapid without compromising the ambition of the review. One example is increasing the resources available for undertaking a review enabling it to be completed quickly. These include the already discussed increased use of automation and the asking for crowd-sourced support in undertaking reviews. Another example is undertaking a review where there are more agreed concepts or using an existing evaluative framework [7]. Reviews of reviews, which are a further meta level of research, can also sometimes provide a quick method of determining what is known from research. These reviews of reviews may provide breadth and overview but their higher meta level and use of already synthesised findings may limit the extent of further synthesis.

The key issue is the fitness for purpose of the methods and resources in their ability to provide useful and justifiable evidence claims in response to the research question. A quick non-systematic scope of a literature may be fit for purpose in planning a systematic review. A rapid review that maintained rigour but achieved rapidity by limiting the review’s scope or increasing resources available may be fit for purpose if it can still provide useful answers to those asking the question. More dangerous is a rapid review that maintains a large ambition but compromises rigour so that any evidence claims are not really justifiable. Due to the potential confusion in this area, it might help if all reviews were clear about their ambition, their rigour, how any stated rapidity is being achieved, and potential limitations to any evidence claims made. Consumers of reviews need to know how a review question has been framed, the methods used to address this question and the claims that then can be made.

### A dynamic temporal approach to reviews

Time is not just an issue in terms of how long a review takes to be completed. It is also relevant to when a review is undertaken and its relation to what is known, what people want to be known, and how this is likely to change. A review provides a statement of an evidence base at a particular point in time. Over time there can be changes in the following:

- The questions being asked by users of research and research producers
- What studies have been undertaken (including administrative data sets)
- What is known from research about various research questions
- The paradigm within which the research is conducted, resulting in shifts in understanding and perception about how results should be interpreted and research questions framed
- Methods and processes (including automation) of research
- The functioning of the evidence ecosystem of which the research is part
- The wider context within which the evidence ecosystem exists

Areas of concern to decision-makers and the research available to inform their decisions will change over time. In a particular topic area, there may be a range of reviews and primary studies for decision-makers and researchers to call upon. Decisions about investing in new reviews or new primary studies at any one time will depend on the questions of concern, the quality and relevance of the reviews already available, the time and other resources available, and the balance between responding to immediate needs and longer-term contributions to developing the evidence base.

In some cases, a very narrowly focused question will be highly relevant and useful for filling an important gap in the evidence base to inform a decision. In other cases, highly context-specific pieces of work may be necessary to complement and interpret a more generic review of research. Similarly, those interested in broad questions examining, for example, a theory of change may undertake a range of different reviews and primary studies at different points of time on different parts of the model to further understanding of how social policies and practices have an effect.

At the other extreme, if the timescale before decisions are to be made are short then rapid reviews maybe very relevant as long as they meet the evidence standards for making justifiable evidence claims. If there is no basis to a claim, then it should not inform decision-making.

The required depth and breadth of any new primary research or review of research depends upon its role at a particular point of time (and over time) within an evidence ecosystem. Research use and research production is a dynamic temporal process where longer-term strategic approaches may be more efficient and so more helpful. This suggests a temporal strategic approach to undertaking and using research. This may include gauging user needs for research, the state of the art and maturity of the research, and how decision-making and research engage together. For those making practice and policy decisions, these decisions may not be single events but part of a process of system improvement over time.

Finally, taking an evidence ecosystem perspective can lead to more efficiency and less waste in planning reviews including a more strategic view of the balance between the needs of decision-makers (including researchers), engagement with research, research synthesis, and primary research.

## Conclusion

There have been many advances in the methods and use of systematic reviews over recent years, but there has been no major progress in creating a coherent classification of review methods. This may not be a problem as long as we continue to pay attention to these differences in planning, reporting, and interpreting reviews. The current paper extends the analysis we published in 2012 by considering the broader context of the evidence ecosystem within which reviews are produced and the way that these are constantly evolving. This broader view of research use and production helps provide an understanding of the contextual factors that underlie the variation in research methods of systematic reviews.
